# Outcomes of t(11;14) light chain (AL) amyloidosis after autologous stem cell transplantation: benchmark for new therapies

**DOI:** 10.1038/s41408-023-00945-0

**Published:** 2023-11-15

**Authors:** Susan Bal, Noel Estrada-Merly, Luciano J. Costa, Muzaffar H. Qazilbash, Shaji Kumar, Anita D’Souza

**Affiliations:** 1 Division of Hematology/Oncology, Department of Medicine, University of Alabama at Birmingham, Birmingham, AL USA; 2 Division of Hematology/Oncology, Department of Medicine, Medical College of Wisconsin, Milwaukee, WI USA; 3 Division of Cancer Medicine, Department of Stem Cell Transplantation, MD Anderson Cancer Center, Houston, TX USA; 4 Division of Hematology, Department of Medicine, Mayo Clinic, Rochester, MN USA

**Keywords:** Cancer, Medical research

**Dear Editor,**

Systemic light chain (AL) amyloidosis is a clonal plasma cell disorder characterized by abnormal immunoglobulin light chain production, which forms insoluble fibrillary aggregates that deposit in tissues causing end-organ dysfunction. Cardiac involvement predominantly drives early mortality and influences patients’ functional status, treatment choice, and prognosis [1]. The management of AL amyloidosis focuses on clone eradication using plasma cell-directed therapies. Autologous stem cell transplant (ASCT) can result in a deep and durable response in carefully selected patients [2].

The underlying clonal plasma cells in AL amyloidosis harbor recurrent cytogenetic abnormalities detected by interphase fluorescence in situ hybridization (iFISH), with t(11;14) being the most frequent (40–50%) [3, 4]. This translocation juxtaposes the IGH gene on chromosome 14 next to the proto-oncogene CCND1 on chromosome 11 resulting in overexpression of cyclin D1. This translocation results in distinct pathologic and clinical features and is predictive of response to therapy. Patients with t(11;14) are less likely to have a favorable hematologic response and event-free survival with proteasome inhibition but tend to respond more favorably to alkylator therapy, including ASCT [5, 6]. Additionally, t(11;14) is associated with a higher dependency of the plasma cell on the anti-apoptotic protein BCL2 and as such, deep responses are reported with BCL2 inhibition using venetoclax in AL amyloidosis in retrospective series [7, 8].

We sought to characterize the outcomes of patients with AL amyloidosis with and without t(11;14) who underwent ASCT to understand if there was a differential response to ASCT by t(11;14) status. We used publicly available, de-identified Center for International Blood and Marrow Transplant Research (CIBMTR) data from a study of AL amyloidosis patients who received upfront ASCT in the United States between 1 January 2014 and 31 December 2018 (*N* = 440) within nine months from initial diagnosis [9, 10]. We selected patients with available cytogenetics information (*N* = 394) and excluded those where cytogenetic information was not available (*N* = 46). We used descriptive statistics to summarize baseline characteristics and tested for differences in continuous variables using Kruskal-Wallis test, and Pearson *χ*^2^ test or Fisher’s exact test for categorical variables. The Kaplan-Meier method with a Log-rank test was used to estimate and compare progression-free survival (PFS) and overall survival (OS) probabilities. For relapse/progression, we used the cumulative incidence function and Gray’s test to account for competing risks. We constructed a multivariate Cox proportional hazards model using a stepwise approach selecting covariates with a 5% cut-off. The following covariates were included in the model-building process: t(11;14) [*main effect*], bortezomib induction, age at transplant, performance status, sex, renal involvement, cardiac involvement, liver involvement, number of organs involved, creatinine at diagnosis, melphalan dose, center experience (<4 vs 4 or more AL amyloidosis transplants/year).

Of the 394 patients, 102 patients had t(11;14). Baseline characteristics are summarized in Table 1. Patients with t(11;14) were more likely to have cardiac involvement (64% versus 48%, *p* 0.02) and higher number of involved organs (75% vs 57% with ≥2 organs, *p* < 0.01) compared to those without t(11;14). More renal insufficiency was seen without t(11;14) compared to with t(11;14) (73% vs. 68%, *P* = 0.03). More patients with t(11;14) received 200 mg/m^2^ melphalan (Mel 200) conditioning with ASCT compared to those without (63% vs 39%, *p* < 0.01).

**Table 1:** Baseline characteristics by t(11;14) status.

Characteristic – median range or no. (%)	t(11;14): absent (*n* = 292)	t(11;14) present (*n* = 102)	P Value
Median age (range)	61.2 (23.5–76.9)	63.2 (38.4–78.3)	0.29^a^
Sex (*n* = 394), Male	159 (54.5)	65 (63.7)	0.10^b^
Race			0.24^b^
White	245 (83.9)	91 (89.2)	–
Black or African American	35 (12.0)	7 (6.9)	–
Other	8 (2.7)	1 (1.0)	–
Unknown	4 (1.4)	3 (2.9)	–
Karnofsky score ≥90 (*n* = 287)	134 (45.9)	46 (45.1)	0.76^b^
HCT-CI			0.32^b^
0	52 (17.8)	22 (21.6)	–
1	36 (12.3)	11 (10.8)	–
2	49 (16.8)	10 (9.8)	–
3+	155 (53.1)	59 (57.8)	–
Cardiac involvement (*n* = 336)	141 (48.3)	65 (63.7)	0.02^b^
Renal involvement (*n* = 314)	212 (72.6)	69 (67.6)	0.03^b^
Liver involvement (*n* = 364)	34 (11.6)	14 (13.7)	0.67^b^
Organ involvement			<.01^b^
1	127 (43.5)	26 (25.5)	–
2	90 (30.8)	45 (44.1)	–
≥3	75 (25.7)	31 (30.4)	–
Serum creatinine at diagnosis <2 mg/dl (*n* = 365)	226 (77.4)	92 (90.2)	0.01^b^
Serum albumin at diagnosis <3.5 g/dL (*n* = 352)	172 (58.9)	57 (55.9)	–
Bone marrow plasma cells at diagnosis			0.07^b^
<10%	178 (61.0)	60 (58.8)	–
≥10%	88 (30.1)	39 (38.2)	–
Missing	26 (8.9)	3 (2.9)	–
Time from diagnosis to transplant - median (min-max)	5.4 (1.0–9.0)	5.7 (1.5–8.8)	0.90^a^
Bortezomib used in induction	203 (69.5)	67 (65.7)	0.47^b^
Melphalan dose in conditioning regimen, mg/m			<.01^b^
MEL100	34 (11.6)	11 (10.8)	–
MEL140	100 (34.2)	16 (15.7)	–
MEL180	45 (15.4)	11 (10.8)	–
MEL200	113 (38.7)	64 (62.7)	–
Year of transplant			0.06^b^
2014	57 (19.5)	18 (17.6)	–
2015	61 (20.9)	25 (24.5)	–
2016	69 (23.6)	24 (23.5)	–
2017	71 (24.3)	14 (13.7)	–
2018	34 (11.6)	21 (20.6)	2018
Follow-up - median (range)	24.8 (3.3–62.8)	25.2 (3.5–59.8)	–

Hypothesis testing:

^a^Kruskal–Wallis test.

^b^Pearson *χ*^2^ test.

**Fig. 1:**
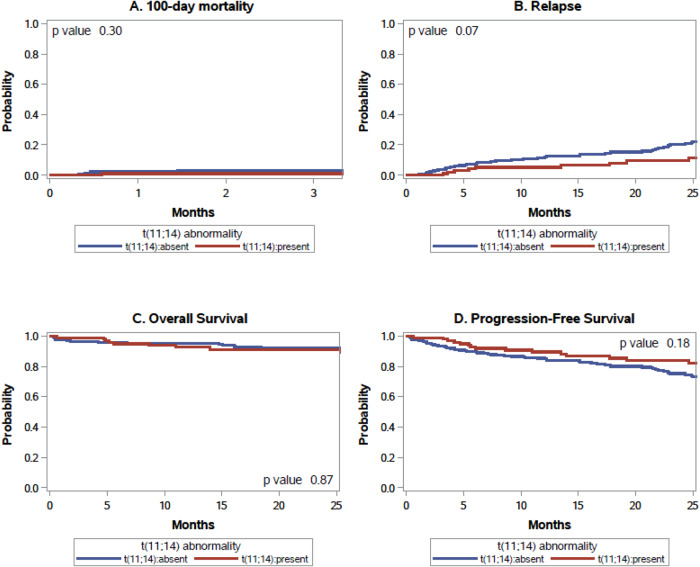
Outcomes of patients after ASCT. **A** Cumulative incidence plot for day 100 mortality by t(11;14) mutation status. **B** Cumulative incidence plot for relapse by t(11;14) mutation status. **C** Kaplan–Meier plot for overall survival by t(11;14) mutation status. **D** Kaplan-Meier plot for progression-free survival by t(11;14) mutation status.

At a median follow up of 25 months (range, 3.3–62.8 months), there was no difference in hematologic complete response rate with 42% in the t(11;14) group versus 35% in the non-t(11;14) group, *p* 0.39. There was no difference in day 100 mortality, hematologic PFS and OS between patients with and without t(11;14) (Fig. 1). A modest, albeit non-significant, trend towards a lower hematologic relapse was seen in patients with t(11;14) compared to those without t(11;14) at 1 year (5.1% vs. 11.3%) and 2 years (9.5% vs. 20.1%) post-ASCT, respectively (*p* 0.067) and similarly, superior PFS was seen in patients with t(11;14) compared to those without t(11;14) at 1 year (90% vs. 81%) and 2 years (84% vs. 71%) post-ASCT, respectively (*p* 0.18). In multivariate analysis, the presence of t(11;14) was not associated with relapse, PFS or OS including in a subset of patients who received higher dose of melphalan conditioning. Factors associated with decreased PFS included omission of induction therapy, KPS < 90, melphalan dose ≤140 mg/m^2^, whereas >70 y, KPS < 90 and serum creatinine ≥2 mg/dl was associated with worse OS. Detailed results are reported in Supplemental Table 1. To address the potential impact of missing t(11;14) status, we performed additional analyses to compare characteristics and outcomes of patients with missing t(11;14) status (*n* = 46). Compared to patients with t(11;14) status available, patients with missing t(11;14) status were more likely to have a missing bone marrow result (35% vs 7%, *p* < 0.01). These patients were also less likely to receive induction treatment (52% vs 69%, *p* 0.03), and had lower HCT-CI score (37% with HCT-CI of 0 compared to 19%, *p* 0.02), with no difference in outcomes (Supplemental table 2). A center effect with centers performing 4 or more ASCT/year for AL amyloidosis being more likely to have greater proportion of patients with t(11;14) as well as using higher doses of melphalan in conditioning.

This is the largest study to describe the outcomes of AL amyloidosis patients with t(11;14) treated with ASCT. Though an effective therapy for AL amyloidosis since the 1990s, due to vital organ involvement and heavy symptom burden, ASCT is only feasible in about 20–25% of patients. Nonetheless, with improvement in patient selection criteria and supportive care over the years, the treatment-related mortality (TRM) has significantly decreased from 20–25% to less than 3% in carefully selected patients as shown in this and other registry studies [2, 9]. The role of ASCT in upfront treatment of AL amyloidosis has been further questioned in recent times given high hematologic responses seen with daratumumab combined with cyclophosphamide, bortezomib, and dexamethasone quadruplet therapy in the ANDROMEDA study, including in those with t(11;14), albeit with short follow up [11, 12].

The presence of t(11;14) is a prognostic and predictive biomarker in AL amyloidosis. This biomarker is associated with higher likelihood of cardiac involvement, also confirmed by our study [4, 13]. The lower hematologic responses and survival when treated with proteasome inhibitors (particularly bortezomib) and immunomodulatory agents are abrogated with the deployment of ASCT for this population as noted by no difference in outcomes by the presence of this translocation [14]. The 2-year PFS and OS for this subgroup 84 and 91%, respectively is similar to those subjects without the translocation. This was previously suggested by smaller studies and comparable in our larger multi-center cohort. We also noted a trend towards numerically lower relapses at one- and two-years following ASCT for this cohort suggesting a greater impact of ASCT in improving outcomes. While the best hematologic complete response rate of 30–40% noted in this study are inferior to the results seen with antiCD38-based monoclonal antibody combinations [12], the determination of efficacy of combinatorial approaches in carefully selected patients in ongoing studies will be key. Furthermore the outcomes of patients with this translocation can be further modulated by the use of targeted therapies that exploit the BCL-2 dependency of this subset and impressive responses are noted in retrospective series of venetoclax in relapsed amyloidosis [7, 8]. Several prospective clinical trials of BCL-2 inhibition in this subset are ongoing (NCT03000660, NCT05451771, NCT04847453, NCT05486481).

Our study has limitations including the inherent drawbacks of a retrospective registry study. Additionally, these outcomes are only applicable to ASCT-eligible AL amyloidosis patients and may not mirror the outcomes of all AL amyloidosis patients, particularly those who are transplant-ineligible. The proportion of patients with t(11;14) in the present study is lower than other reported studies and is likely due to this being a transplant study and patients with t(11;14) present having more advanced disease with greater cardiac involvement are less likely to be transplant-eligible. It is also possible that the lower proportion of t(11;14) noted could be due to false negative results, especially if CD138 enrichment is not performed. These data, which are available until 2018, are prior to the approval and subsequent use of anti-CD38 monoclonal antibody-based combinations and prolonged therapy without routine ASCT.

In conclusion, we report the outcomes of ASCT in AL amyloidosis patients showing that AL amyloidosis patients with t(11;14) have similar outcomes to those without t(11;14). These results can serve as a benchmark for the further characterization of outcomes of this unique subset of patients in the context of quadruplet therapy and anti-BCL2 therapies both with and without ASCT in the future.

### Supplementary information

Supplemental tables
