# Supplementary material for: Outcomes of t(11;14) light chain (AL) amyloidosis after autologous stem cell transplantation: benchmark for new therapies
Source: Blood Cancer J. 2023 Nov 15;13(1):170. doi: 10.1038/s41408-023-00945-0 (PMC10651880; doi:10.1038/s41408-023-00945-0)
Supplement: Supplementary file 1 — Supplemental tables [file 41408_2023_945_MOESM1_ESM.docx]

Supplementary Table 1. Multivariate Analysis. Covariates used in model building include t11:14 (main effect), Bortezomib induction, age at transplant, KPS, sex, renal involvement, cardiac involvement, liver involvement, number of organs involved, creatinine at diagnosis, melphalan dose, center experience.

| **Covariate** | **Hazard Ratio (95% CI)** | **p-value** |
| --- | --- | --- |
| *Overall Survival* |  |  |
| t(11;14) status (Main effect) |  |  |
| t(11;14) absent | Reference | 0.57 |
| t(11;14) present | 1.25 (0.59-2.66) | 0.57 |
| KPS |  |  |
| 90-100 | Reference | 0.005 |
| <90 | 3.05 (1.40-6.66) | 0.005 |
| Renal involvement |  |  |
| No | Reference | 0.026 |
| Yes | 1.39 (0.31-6.12) | 0.67 |
| Not done/Missing | 3.73 (0.79-17.62) | 0.097 |
| Creatinine at diagnosis |  |  |
| <2mg/dl | Reference | 0.0207 |
| >=2mg/dl | 2.59 (1.16-5.79) | 0.0207 |
| Age at AHCT |  |  |
| <50 | Reference | 0.0001 |
| 50-59 | 1.18 (0.24-5.72) | 0.8416 |
| 60-69 | 2.57 (0.59-11.29) | 0.2103 |
| 70+ | 9.01 (1.91-42.49) | 0.0054 |
| *Progression-free Survival* |  |  |
| t(11;14) status (Main effect) |  |  |
| t(11;14) absent | Reference | 0.7693 |
| t(11;14) present | 0.93 (0.57-1.52) | 0.7693 |
| Bort induction |  |  |
| No | Reference | 0.036 |
| Yes | 0.64 (0.42-0.97) | 0.036 |
| KPS |  |  |
| 90-100 | Reference | 0.02 |
| <90 | 1.74 (1.12-2.72) | 0.02 |
| Melphalan conditioning dose, mg/m2 |  |  |
| MEL200 | Reference | 0.0005 |
| MEL100 | 2.30 (1.20-4.41) | 0.01 |
| MEL140 | 2.83 (1.69-4.71) | <.0001 |
| MEL180 | 1.22 (0.61-2.45) | 0.58 |
| *Relapse* |  |  |
| t(11;14) status (Main effect) |  |  |
| t(11;14) absent | Reference | 0.14 |
| t(11;14) present | 0.64 (0.36-1.15) | 0.14 |
| Bort used in induction |  |  |
| No induction | Reference | 0.003 |
| Bort induction | 0.50 (0.32-0.79) | 0.003 |
| Melphalan conditioning dose, mg/m2 |  |  |
| MEL200 | Reference | 0.005 |
| MEL100 | 2.80 (1.42-5.54) | 0.003 |
| MEL140 | 2.14 (1.25-3.68) | 0.006 |
| MEL180 | 1.04 (0.49-2.23) | 0.92 |
| KPS |  |  |
| 90-100 | Reference | 0.0039 |
| <90 | 2.02 (1.25-3.26) | 0.0039 |
| **SUBSET ANALYSIS OF MEL 180/200 (N= 233)** | | |
| **Covariate** | **Hazard Ratio (95% CI)** | **p-value** |
| *Overall Survival* |  |  |
| t(11;14) status (Main effect) |  |  |
| t(11;14) absent | Reference | 0.67 |
| t(11;14) present | 0.77 (0.24-2.52) | 0.67 |
| KPS |  |  |
| 90-100 | Reference | 0.01 |
| <90 | 6.75 (1.50-30.50) | 0.01 |
| *Progression-free Survival* |  |  |
| t(11;14) status (Main effect) |  |  |
| t(11;14) absent | Reference | 0.81 |
| t(11;14) present | 0.92 (0.47-1.82) | 0.81 |
| Cardiac involvement |  |  |
| No | Reference | 0.04 |
| Yes | 1.84 (0.93-3.66) | 0.08 |
| Not Done/Missing | 0.37 (0.08-1.62) | 0.19 |
| *Relapse* |  |  |
| t(11;14) status (Main effect) |  |  |
| t(11;14) absent | Reference | 0.31 |
| t(11;14) present | 0.67 (0.31-1.44) | 0.31 |
| KPS |  |  |
| 90-100 | Reference | 0.02 |
| <90 | 2.23 (1.12-4.42) | 0.02 |

Supplementary table 2. Sensitivity analysis by t(11;14) missing status.

| **Characteristic –**  **median range or no. (%)** | | | **t(11;14): information available (n=394)** | | | | **Not done, missing (n=46)** | | **P Value** | | |
| --- | --- | --- | --- | --- | --- | --- | --- | --- | --- | --- | --- |
| Median age (range) - median (min-max) | | | 61.5 (23.5-78.3) | | | | 60.8 (33.9-71.6) | | 0.51**^a^** | | |
| Male gender (n= 440) | | | 224 (56.9) | | | | 24 (52.2) | | 0.54**^b^** | | |
| Race | | |  | | | |  | | 0.28**^b^** | | |
| White | | | 336 (85.3) | | | | 37 (80.4) | |  | | |
| Black or African-American | | | 42 (10.7) | | | | 6 (13.0) | |  | | |
| Other | | | 9 (2.3) | | | | 3 (6.5) | |  | | |
| Unknown | | | 7 (1.8) | | | | 0 (0.0) | |  | | |
| Karnofsky score >= 90 (n= 431) | | | 180 (45.7) | | | | 20 (43.5) | | 0.50**^b^** | | |
| HCT-CI | | |  | | | |  | | 0.02**^b^** | | |
| 0 | | | 74 (18.8) | | | | 17 (37.0) | |  | | |
| 1 | | | 47 (11.9) | | | | 3 (6.5) | |  | | |
| 2 | | | 59 (15.0) | | | | 8 (17.4) | |  | | |
| 3+ | | | 214 (54.3) | | | | 18 (39.1) | |  | | |
| Cardiac involvement (n= 374) | | | 206 (52.3) | | | | 19 (41.3) | | 0.37**^b^** | | |
| Renal involvement (n= 350) | | | 281 (71.3) | | | | 31 (67.4) | | 0.81**^b^** | | |
| Liver involvement (n= 405) | | | 48 (12.2) | | | | 6 (13.0) | | 0.72**^b^** | | |
| Organ involvement | | |  | | | |  | | 0.55**^b^** | | |
| 1 | | | 153 (38.8) | | | | 19 (41.3) | |  | | |
| 2 | | | 135 (34.3) | | | | 18 (39.1) | |  | | |
| >=3 | | | 106 (26.9) | | | | 9 (19.6) | |  | | |
| Serum creatinine at diagnosis < 2 mg/dl  (n= 407) | | | 318 (80.7) | | | | 40 (87.0) | | 0.30**^b^** | | |
| Serum albumin at diagnosis < 3.5 g/dL  (n= 392) | | | 229 (58.1) | | | | 25 (54.3) | | 0.84**^b^** | | |
| Bone marrow plasma cells at diagnosis | | |  | | | |  | | <.01**^b^** | | |
| <10% | | | 238 (60.4) | | | | 25 (54.3) | |  | | |
| >=10% | | | 127 (32.2) | | | | 5 (10.9) | |  | | |
| Missing | | | 29 (7.4) | | | | 16 (34.8) | |  | | |
| Time from diagnosis to transplant - median (min-max) | | | 5.5 (1.0-9.0) | | | | 4.8 (0.7-8.8) | | 0.35**^a^** | | |
| Bort used in induction | | | 270 (68.5) | | | | 24 (52.2) | | 0.03**^b^** | | |
| Melphalan dose in conditioning regimen, mg/m | | |  | | | |  | | 0.28**^b^** | | |
| MEL100 | | | 45 (11.4) | | | | 3 (6.5) | |  | | |
| MEL140 | | | 116 (29.4) | | | | 9 (19.6) | |  | | |
| MEL180 | | | 56 (14.2) | | | | 8 (17.4) | |  | | |
| MEL200 | | | 177 (44.9) | | | | 26 (56.5) | |  | | |
| Year of transplant | | |  | | | |  | | 0.54**^b^** | | |
| 2014 | | | 75 (19.0) | | | | 7 (15.2) | |  | | |
| 2015 | | | 86 (21.8) | | | | 8 (17.4) | |  | | |
| 2016 | | | 93 (23.6) | | | | 13 (28.3) | |  | | |
| 2017 | | | 85 (21.6) | | | | 8 (17.4) | |  | | |
| 2018 | | | 55 (14.0) | | | | 10 (21.7) | |  | | |
| Follow-up - median (range) | | | 25.0 (3.3-62.8) | | | | 23.8 (3.4-49.0) | |  | | |
| Hypothesis testing: **^a^** Kruskal-Wallis test **^b^** Pearson chi-square test | | | | | | | | | | | |
|  | **t(11;14) absent**  **(N = 292)** | | | **t(11;14) present**  **(N = 102)** | | **Test not done/Unknown**  **(N = 46)** | | | |  |  |
| **Outcomes** | **N** | **Prob (95% CI)** | | **N** | **Prob (95% CI)** | **N** | | **Prob (95% CI)** | | **P Value** |  |
| Day 100 mortality | 286 |  | | 101 |  | 46 | |  | | 0.43 |  |
| 100-day |  | 2.8 (1.2-5)% | |  | 1 (0-3.9)% |  | | 4.3 (0.4-12.2)% | |  |  |
| Relapse | 286 |  | | 101 |  | 46 | |  | | 0.13 |  |
| 1-year |  | 11.3 (7.8-15.4)% | |  | 5.1 (1.6-10.3)% |  | | 12.1 (3.9-24)% | |  |  |
| 2-year |  | 20.1 (15.1-25.6)% | |  | 9.5 (4.1-16.9)% |  | | 12.1 (3.9-24)% | |  |  |
| Progression free survival | 286 |  | | 101 |  | 46 | |  | | 0.42 |  |
| 1-year |  | 85.5 (81.1-89.4)% | |  | 89.6 (82.7-94.9)% |  | | 81.1 (68-91.4)% | |  |  |
| 2-year |  | 75.3 (69.6-80.7)% | |  | 84 (75.5-91)% |  | | 77.1 (62.2-89.1)% | |  |  |
| Overall survival | 292 |  | | 102 |  | 46 | |  | | 0.10 |  |
| 1-year |  | 95.1 (92.3-97.3)% | |  | 92.7 (86.7-97)% |  | | 90.5 (79.8-97.4)% | |  |  |
| 2-year |  | 92.1 (88.4-95.1)% | |  | 91.3 (84.6-96.2)% |  | | 82.3 (67-93.5)% | |  |  |
